# Learning from living: chance, curiosity and colleagues

**DOI:** 10.1113/EP092460

**Published:** 2024-12-20

**Authors:** Hugh Montgomery

**Affiliations:** ^1^ Centre for Human Health and Performance University College London London UK

Even without the temporal distortion that *anno domini* brings, I am now aware that I am accelerating towards the event horizon at pace. A good time, then, to reflect on lessons learned in life and, specifically, in a life of clinical physiology and pathophysiology.

In 1981, I started at the Middlesex Hospital Medical School. Eric Neil (honorary treasurer of the Physiological Society and President of the International Union of Physiological Sciences) was Professor of Physiology. Initially, I found his first lecture terrifying and bewildering; a collection of mumbled signposts, anecdotes and descriptions of past experiments. I scurried back to interrogate ‘*Samson Wright's Applied Physiology*’ (of which he was editor), only to be kicked out of the second lecture ‘for knowing too much’. It was a complement. He took me under his wing, pushed me hard and harder still, and was a mentor until the day he died. He left me an old framed Bournville Chocolate advert from a London Underground train, with a Viking facing the bow in a dragon‐prowed longboat. He'd signed it, ‘Follow the direction of the dragon's tongue, and you'll not go far wrong’. He wasn't the last to tell me to be self‐propelled. A lesson learned.

My clinical path led me to intensive care medicine. In 1988, when I started at the Hammersmith Hospital, there was no such speciality, but it did not take me long to realize that every patient was one giant ‘physiological preparation’. Amongst others, I worked for the wonderful (Professor Sir) Colin Dollery, from who I also learned a great deal. Coming to see his ‘on take’ emergency admissions at 04.00 h (he was flying to China at 08.00 h), I took him to a drunk alcoholic diabetic who had taken an overdose. I had dealt with all the issues perfectly, but had not performed retinoscopy (to look for background diabetic changes) for lack of time. My management was, he told me, ‘suboptimal’. He was right. He could be bothered to have a sleepless night for the benefit of the patients. I should not have skimped. Another lesson learned.

I had no intention of going into research as a career, but (in those days) needed to ‘tick the box’ by gaining a higher degree were my career to proceed. I started out studying the role of the renin–angiotensin system (RAS) in driving vascular smooth muscle cell growth and soon found that things were not working as I had wished. Well over a year in, I was in despair when (recently retired Professor of Pharmacology in Cardiff) Gary Baxter took me to one side. ‘Plough your own furrow’, he said. I thought of Eric. I did as they both suggested.

Just as I had found that ‘being bothered’ to read more had helped me at medical school, so too did this apply now. Data were beginning to appear which suggested that the RAS played a role in cardiac growth, probably by synthesizing angiotensin II, at least in vitro, in pigs and in rodents (Baker et al., 1992; Fernandez‐Alfonso et al., 1992). But what about humans? A paper appeared which identified a human genetic variant of the human angiotensin‐converting enzyme gene (*ACE*), the ‘D’ allele being associated with higher circulating ACE activity than the equally common ‘I’ allele (Rigat et al., 1990), with the same later shown to apply in heart tissue. If the RAS regulated human cardiac growth, then cardiac mass ought to be related to *ACE* genotype. The problem was that too many environmental factors drive heart growth (blood pressure, exercise, diabetes and heart valve disease), and greater growth in such circumstances is associated with increased risk of death. How, then, to explore the question?

I had taken up running by then. Eric Neil started me off, having been an elite fell runner. A routine ECG had shown me to have cardiac hypertrophy (within normal limits), probably owing to the increased cardiac load. This chance finding prompted the answer. I would study the cardiac hypertrophic response to short‐term exercise training. Specifically, I would study army recruits: same sex and mostly race (in those days), similar age, eating the same food, drinking the same water, wearing identical clothing, and doing the same 10 weeks of exercise training. Differences in cardiac growth would thus be strongly genetically determined. And so it turned out! The *ACE* D‐allele was strongly associated with cardiac growth (Montgomery et al., 1997), an effect we later confirmed and showed be dependent on ACE‐related regulation of bradykinin activity (Brull et al., 2001; Myerson et al., 2001). On a hunch, I guessed that if cardiac and vascular smooth muscle cell growth was RAS dependent, then so too would be that of skeletal muscle, which turned out also to be correct (Montgomery et al., 1999).

However, presenting the data one day, I was challenged from the audience. Surely the ‘DD genotype’ individuals might simply have been less fit than those with the ‘II genotype’, and they had to do more cardiac work as a result (thus driving greater cardiac and skeletal muscle growth)? The idea was clearly nonsense (to my mind), but I set out to do a quick experiment to prove it and nail further criticism. I was wrong. Improvements in performing repetitive loaded biceps curls over 10 weeks of general physical training was I‐allele related, and the I‐allele was over‐represented in elite high‐altitude mountaineers (Montgomery et al., 1998). Muscle exertional metabolic efficiency was indeed *ACE* genotype dependent, with improvements in ‘delta efficiency’ with army training also being I‐allele dependent (Williams et al., 2000). We went on to show that the D‐allele was indeed associated with power‐related sports and the I‐allele with endurance (Myerson et al., 1999; Tsianos et al., 2004; Woods et al., 2001, 2000).

By now, running and walking in the hills had been replaced by mountaineering, and I was offered the place of Medical Officer on an expedition to climb the south‐east face of Pumori (7161 m a.s.l.) in the Himalaya. I had never been that high before, and I was breathless as I slogged for 4 h between Pheriche (4371 m a.s.l.) and Goarekshep (5140 m a.s.l.). No surprises there! The partial pressure of oxygen there is half that at sea level, and the primary adaptation to hypoxia (I had been taught) was to increase delivery and extraction (higher heart rate, higher respiratory rate, slowly rising red cell mass and more mitochondria). I certainly could not have had a higher respiratory or cardiac rate. Dawn, however, saw an emergency in a trekker, and I had to get her to safety, and fast. I agreed to return that night and head on to join the team at the next camp. However, once I had delivered her to the makeshift medical facility at Pheriche, the sun was getting low and bad weather appeared to be coming in. I set out and made the same ascent as the day before, but in merely 40 min. There was no way that this change in performance could have been owing to greater ventilatory effort, cardiac work or haemoglobin availability. But what was it?

Serendipity can help to give answers, sometimes. Not long after my return, I limped onto a stage at Great Ormond Street Hospital to present my RAS data, having grunted my way through a marathon 2 days earlier. The speaker after me talked of how cooling the heads of sick infants could be neuroprotective by lowering metabolic rate. Of course! Hypoxic adaptation was not driven by increasing oxygen delivery; it was mediated by changing metabolism and its efficiency! We hastily convened a round table to explore the idea. Held in a small hotel in Windsor, we called it ‘Give and Take’. Many of the great and good came, including John West. The idea seemed to ‘have legs’. It would account for why the I‐allele was associated with greater metabolic efficiency and also endurance.

If the I‐allele was associated with metabolic efficiency and endurance performance at sea level, how much greater might that effect be at high altitude, where oxygen was scarce? The answer was, ‘a lot’ (Kalson et al., 2009; Montgomery et al., 1998; Thompson et al., 2007; Tsianos et al., 2005, 2004). But how to explore this further? In the Himalaya, I had been astonished at the extraordinary ability of native Tibetans (whose ancestors were lowlander Han Chinese) to perform at altitude. Natural selection seemed at work, so I headed to collect DNA samples across an altitude cline through China, from the lowlands to the Tibetan border. (A team also collected mouse DNA in the Andes and across altitude gradients in the Andes and on Hawaii, but the samples were accidentally thrown away by our US collaborator.) Human genetic analysis was in its infancy at the time, so it was only many years later that we performed a genome‐wide association study (using Han DNA as a control) to find the genes selected. Meanwhile, however, we showed mitochondrial coupling to be regulated by ACE (Dhamrait et al., 2016).

I was now a practising intensive care doctor (albeit in training), and greatly bemused. Seemingly identical patients (age, sex, race, disease and disease severity) who received the same treatment could have totally different outcomes. Some lived, and some died. We would always pore over our management. What magic element had we got right there? What had we slipped up on there? Many of our patients suffered from acute (adult) respiratory distress syndrome (ARDS), which left them with profoundly low saturations of arterial blood with oxygen. The same issue of low tissue oxygen delivery seemed to affect many more (whether from anaemia or from poor cardiac or microvascular function). If success in climbing high was *ACE* genotype dependent, could it be that surviving ARDS was similarly predicated? It was (Marshall et al., 2002), and so, too, was outcome in critically ill premature babies (Harding et al., 2003) and in children with meningococcal sepsis (Harding et al., 2002).

Studying hypoxic adaptation in patients is hard, however, owing to the great diversity in patient demographics, disease states and severity, and treatment alluded to above. Much as the answer to studying pathological cardiac hypertrophy was to study physiological hypertrophy, the answer here was to study the response of otherwise healthy individuals exposed to the hypoxia of high altitude. A one‐in‐a‐generation leader, Mike Grocott, thus conceived and delivered the Caudwell Xtreme Everest Expedition of 2007 and its allied high‐altitude research programmes (Grocott et al., 2016). These confirmed that human hypoxic adaptation did seem to be metabolic in origin. Not long afterwards, genetic testing caught up with our hypothesis, and we were able to show that natural selection had indeed been at work in Tibetans, with the most selected ‘high‐altitude’ variant being associated with lower haemoglobin concentrations (Beall et al., 2010). Professor Andy Murray and his group took the idea to far greater heights (pun intended), by looking at mitochondrial function at altitude. He showed that Sherpas (Tibetans) were genetically selected to ‘burn’ a more efficient fuel (glucose over fat) (Horscroft et al., 2017; Murray et al., 2018).

But we had also noted other epiphenomena when climbing. On the Everest (and allied) trips, we all lost weight, despite ready access to high‐quality food in copious quantities. This phenomenon was, of course, well known amongst climbers. We kept food diaries on our 2006 Cho Oyu (8201 m a.s.l.) preparatory trip pre‐Everest, and I was bewildered to find that I had scored my satiety at 9/10 for 2 days (being convinced that I had been eating really well), whereas my food diary showed that I had shared half a Snickers Bar in this time. Furthermore, the loss was of both fat and muscle. This phenomenon was always considered a pathophysiological dysregulation. But could it instead be adaptive? After all, the metabolic cost of protein synthesis is very high (Reeds et al., 1985).

Back to our intensive care unit patients! It was also clear that they, too, lost a huge amount of muscle. This was also considered pathological, although efforts to try to reverse this loss had been shown to be harmful (Takala et al., 1999). Could that, too, be part of a metabolic adaptation? So it seemed! Far from being some pathological effect of critical illness, muscle wasting seemed highly orchestrated at the molecular level; muscle protein synthesis and anabolic pathways were suppressed and catabolic pathways activated (Puthucheary et al., 2013). Further work showed this indeed to be driven by activation of the master regulator of hypoxic sensing, the hypoxia inducible factors (Puthucheary et al., 2018). We had been right all along! Hypoxic adaptation was indeed largely metabolic, and the same pathways were activated in the critically ill. In both circumstances, ‘the obvious answer’ was the right one; when faced with a shortage of ‘metabolic substrate’ (oxidized fuel sources), the response of the body is to use/demand less, rather than to deliver more. As it happened, however, we were only right in part. Network analysis showed that hypoxia inducible factor activation was being driven by inflammatory cytokines and lactate. Another lesson: we should ignore the ‘functional names’ given to some molecules (‘tumour necrosis factor’ does so much more than its name suggests). ‘Hypoxia inducible factors’ are probably better referred to as ‘metabolic demand and supply sensors’.

Thus, lived experience (one's own and that of others) tells one a great deal, so long as one listens and questions. We can all do that. Serendipity also plays a part; the people we meet, the observations we make in sickness and in health. But serendipity is also, to some degree, also under our own control. We can choose to say, ‘yes’ to new experiences and to meeting people, or not. We can choose how hard to push our intellects. And we can choose to surround ourselves with bright, capable, curious and driven people who are our betters. And when knowledge, chance, curiosity and colleagues collide, there is much to be learned.

## AUTHOR CONTRIBUTIONS

Sole author.

## CONFLICT OF INTEREST

None declare.

## FUNDING INFORMATION

Hugh's salary is supported by the National Institute for Health Research's Comprehensive Biomedical Research Centre at University College London Hospitals.
